# Molecular Dynamics Simulation of the Thermosensitive Gelation Mechanism of Phosphorylcholine Groups-Conjugated Methylcellulose Hydrogel

**DOI:** 10.3390/gels11070521

**Published:** 2025-07-04

**Authors:** Hongyu Mei, Yaqing Huang, Juzhen Yi, Wencheng Chen, Peng Guan, Shanyue Guan, Xiaohong Chen, Wei Li, Liqun Yang

**Affiliations:** 1 Institute of Polymer and Material Science, School of Chemistry, Sun Yat-sen University, Guangzhou 510275, China; 2 Instrumental Analysis & Research Center, Sun Yat-sen University, Guangzhou 510275, China; 3 Key Laboratory for Polymeric Composite and Functional Materials of Ministry of Education, Guangdong Provincial Key Laboratory for High Performance Polymer-Based Composites, Institute of Green Chemistry and Molecular Engineering, Sun Yat-sen University, Guangzhou 510275, China

**Keywords:** thermosensitive hydrogel, phosphorylcholine groups-conjugated methylcellulose hydrogel, molecular dynamics simulation, gelation mechanism

## Abstract

The intelligently thermosensitive 2-methacryloyloxyethyl phosphorylcholine (MPC) groups-conjugated methylcellulose (MC) hydrogel, abbreviated as MPC-g-MC, exhibits good potential for prevention of postoperative adhesions. However, its thermosensitive gelation mechanism and why the MPC-g-MC hydrogel shows a lower gelation temperature than that of MC hydrogel are still unclear. Molecular dynamics (MD) simulation was thus used to investigate these mechanisms in this work. After a fully atomistic MPC-g-MC molecular model was constructed, MD simulations during the thermal simulation process and at constant temperatures were performed using GROMACS 2022.3 software. The results indicated that the hydrophobic interactions between the MPC-g-MC molecular chains increased, the interactions between the MPC-g-MC molecular chains and H_2_O molecules decreased with the rise in temperature, and the hydrogen bonding structures were changed during the thermal simulation process. As a result, the MPC-g-MC molecular chains began to aggregate at about 33 °C (close to the gelation temperature of 33 °C determined by the rheological measurement), bringing about the formation of the MPC-g-MC hydrogel in the macroscopic state. The mechanism of MPC-g-MC hydrogel formation showed that its lower gelation temperature than that of the MC hydrogel is attributed to the increase in the interactions (including hydrophobic interactions, hydrogen bonding interactions, Van der Waals and Coulomb forces) induced by the side MPC groups of MPC-g-MC molecules. The thermosensitive gelation mechanism revealed in this study provides an important reference for the development of novel thermosensitive MC-derived hydrogels with gelation temperatures close to human body temperature.

## 1. Introduction

Thermosensitive hydrogels can intelligently transfer from sols to gels in response to temperature changes in the circumstance [1,2]. They thus exhibit promise for applications in the biomaterial field, such as drug- or cell-loaded thermosensitive hydrogels for improving therapeutic efficacies through gelation in specific locations of the body after in vitro injection [3,4,5], constructing tissue engineering scaffolds [6,7], mimicking the extracellular matrix environment [8], and serving as bio-interface coatings to regulate cell adhesion [9].

Methylcellulose (MC) is a cellulose derivative obtained by methylation of plant-derived cellulose, with a typical degree of methyl substitution ranging from 1.4 to 2.0 [10,11]. Aqueous MC solutions exhibit temperature sensitivity at concentrations of approximately 1.5–5 wt%, showing a typical lower critical solution temperature (LCST) behavior [12,13]. That is, MC solutions may exhibit the sol-state at a temperature lower than their gelation temperature, and then transfer to the gel-state at a temperature higher than their gelation temperature. The mechanism behind this LCST behavior is generally considered to be attributed to strong hydrogen bonding between MC chains and H_2_O molecules at low temperatures; when the temperature rises above the gelation temperature, hydrophobic interactions between -CH_3_ groups of MC chains become more dominant, while hydrogen bonding between MC chains and H_2_O molecules weakens, leading to MC chain aggregation and the formation of a three-dimensional physical network, exhibiting a macroscopic sol–gel transition [14,15,16]. MC hydrogels not only possess thermo-sensitivity but also excellent biocompatibility and biodegradability [17], making them suitable for widespread use in many fields, e.g., drug delivery [18], cell culture [19], and as food thickeners [20]. However, the higher gelation temperature of MC hydrogels in the range of 50–70 °C limits their utility at body temperature in biomedical applications [21,22,23].

To reduce the gelation temperature of MC hydrogels, several strategies have been explored, including grafting side chains on MC, blending MC with organic molecules, and modulation via salt effects [24,25,26]. For example, Morozova et al. grafted poly(ethylene glycol) (PEG) chains onto MC, reducing the gelation temperature to approximately 35 °C [24]. Organic compounds, such as propylene glycol or urea, have been shown to alter the polarity and hydrogen bonding networks of an MC solution, resulting in lowering the gelation temperature of the MC hydrogel [25]. Inorganic salts, such as NaCl and CaCl_2_, can also reduce the gelation temperature of MC hydrogels by enhancing hydrophobic aggregation of MC chains and disrupting the hydrogen bonding network between the -OH groups of MC chains and the surrounding H_2_O molecules [26].

2-Methacryloyloxyethyl phosphorylcholine (MPC) is a zwitterionic compound known for its strong hydration capacity due to its ability to form stable hydrogen bond networks with surrounding H_2_O molecules [27]. This structure grants it excellent anti-fouling and bioinert properties by resisting non-specific protein adsorption and cell adhesion [28,29]. Such a unique structure and properties endow MPC-based biomaterials with rapid development, such as blood contacting materials (e.g., coatings of vascular grafts and polymeric heart valves) [30,31], anti-adhesion materials [32], ophthalmic devices (e.g., contact lenses) [33], tissue engineering scaffolds [34], and drug delivery systems [35].

Recently, MPC-based polymer hydrogels with anti-fouling properties and biocompatibility have received much attention, since they showed good application prospects in postoperative anti-adhesion therapy [36,37]. However, these hydrogels lacked a thermosensitive property with a transformation from sols to gels around the body temperature, bringing about difficulty during the injection process.

In our previous work [38], an MPC-grafted methylcellulose derivative (MPC-g-MC) hydrogel was prepared, and its thermosensitive gelation behaviors were studied via rheological measurements and then its gelation temperature was determined to be around 33 °C (close to body temperature). Furthermore, in vivo experiments demonstrated its efficacy in preventing postoperative adhesions when injected into the damaged ceca of Sprague–Dawley rats [38]. However, the thermosensitive gelation mechanism of the MPC-g-MC hydrogel is still unclear. Furthermore, why the MPC-g-MC hydrogel shows a lower gelation temperature than that of the MC hydrogel needs to be intensively studied.

Molecular dynamics (MD) simulation is a powerful tool that provides atomic-scale spatial and temporal resolution for investigating conformational evolution, solvation structure changes, and various non-bonded interactions (e.g., Van der Waals forces, electrostatic forces, and hydrogen bonding) [39]. Herein, in order to elucidate the thermosensitive gelation mechanism of the MPC-g-MC hydrogel and understand the reason why the MPC-g-MC hydrogel could reduce the gelation temperature close to body temperature, the thermosensitive gelation behaviors and characteristics of the MPC-g-MC and MC hydrogels were studied by using MD simulation at the molecular level. Firstly, the MPC-g-MC/H_2_O system was constructed, and then the MD simulations were performed at different temperatures to analyze the interaction mechanisms, aggregation behaviors, and structural transitions during the heating process. Secondly, the MC/H_2_O system was analyzed using similar MD simulations to disclose the effect of the MPC groups on the gelation temperature and behavior of the MPC-g-MC hydrogel.

## 2. Results and Discussion

### 2.1. Thermosensitive Gelation Mechanism of MPC-g-MC Hydrogel

**Figure 1:**
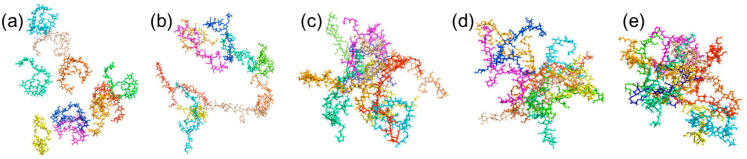
Conformations and aggregation behaviors of MPC-g-MC chains during the heating simulation process (each MPC-g-MC chain was distinguished using different colors): (**a**) 25 °C, (**b**) 30 °C, (**c**) 33 °C, (**d**) 64 °C, (**e**) 80 °C.

The conformation and aggregation behaviors of MPC-g-MC chains during the sol-gel transition process were investigated using MD heating simulation (Figure 1). The MPC-g-MC chains exhibited a loosely dispersed conformation at 25 °C (Figure 1a), corresponding macroscopically to a sol state [38]. As the temperature gradually increased, the MPC-g-MC chains begin to approach and entangle. A noticeable trend of aggregation appeared around 30 °C (Figure 1b), and by approximately 33 °C, the MPC-g-MC chains had aggregated into a compact structure (Figure 1c), indicating a transition to a gel-like state. This aggregated state was maintained stably at 64 °C and 80 °C (Figure 1d,e). The simulated gelation temperature of ~33 °C aligns well with our previous rheological measurements (~33 °C), confirming the reliability of the MD approach.

**Figure 2:**
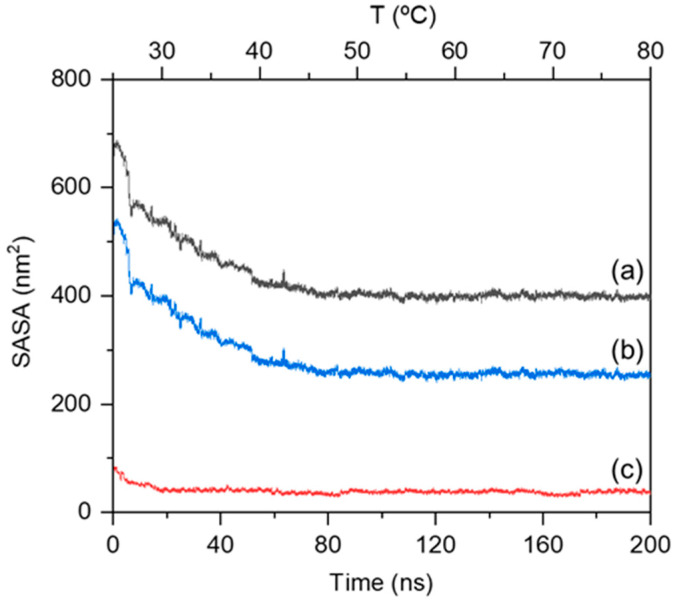
SASA values of MPC-g-MC chains during the heating simulation process: (**a**) total SASA (black curve), (**b**) hydrophobic SASA (blue curve), (**c**) hydrophilic SASA (red curve).

To further investigate the thermodynamic driving forces of chain aggregation, changes in solvent accessible surface area (SASA) along with temperature and time were analyzed in the MPC-g-MC/H_2_O system (Figure 2). The results indicated that total SASA decreased continuously from 25 °C to approximately 80 °C (Figure 2a). Additionally, the downward trend of the hydrophobic SASA curve was more obvious than that of the hydrophilic SASA curve during the heating simulation process (Figure 2b,c). This result indicates that hydrophobic segments of the MPC-g-MC molecules tend to avoid the aqueous environment upon heating, becoming buried in the interior of the aggregate, highlighting hydrophobic interactions as the major thermodynamic driver of gelation.

**Figure 3:**
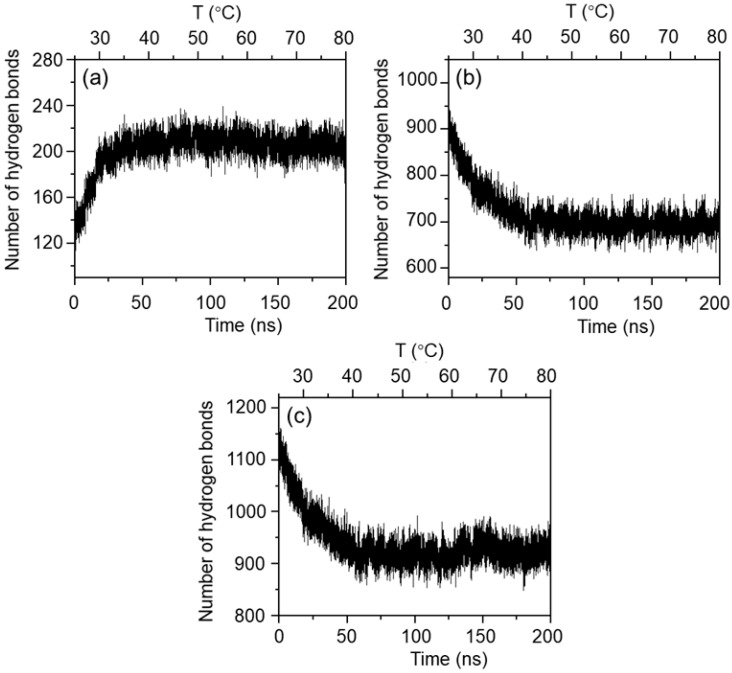
Number of hydrogen bonds in the MPC-g-MC/H_2_O system during the heating simulation process: (**a**) hydrogen bonds between MPC-g-MC chains, (**b**) hydrogen bonds between MPC-g-MC chains and H_2_O molecules, (**c**) total hydrogen bonds in the MPC-g-MC/H_2_O system.

Hydrogen bonding interactions play a dual role in the MPC-g-MC/H_2_O system: the hydrogen bonds between MPC-g-MC chains and the H_2_O solvent can maintain the stability of the aqueous system, whereas the hydrogen bonds between MPC-g-MC chains promotes their aggregation. It should be noted that hydrogen bonding interactions play an important role during the sol–gel transition of the MPC-g-MC hydrogel. Figure 3 shows the changes in the numbers of hydrogen bonds during the heating simulation process. The hydrogen bonds between MPC-g-MC chains increased by ~100 (Figure 3a), while the hydrogen bonds between MPC-g-MC chains and H_2_O molecules decreased by ~200 (Figure 3b), leading to an overall decrease in total hydrogen bonds (Figure 3c). These results suggest that the rise in temperature may promote the formation of new hydrogen bonds between MPC-g-MC chains while destroying the hydrogen bonds between MPC-g-MC chains and H_2_O molecules, bringing about the aggregation of MPC-g-MC chains. This is consistent with the conformation and aggregation behaviors of MPC-g-MC chains, as shown in Figure 1.

**Figure 4:**
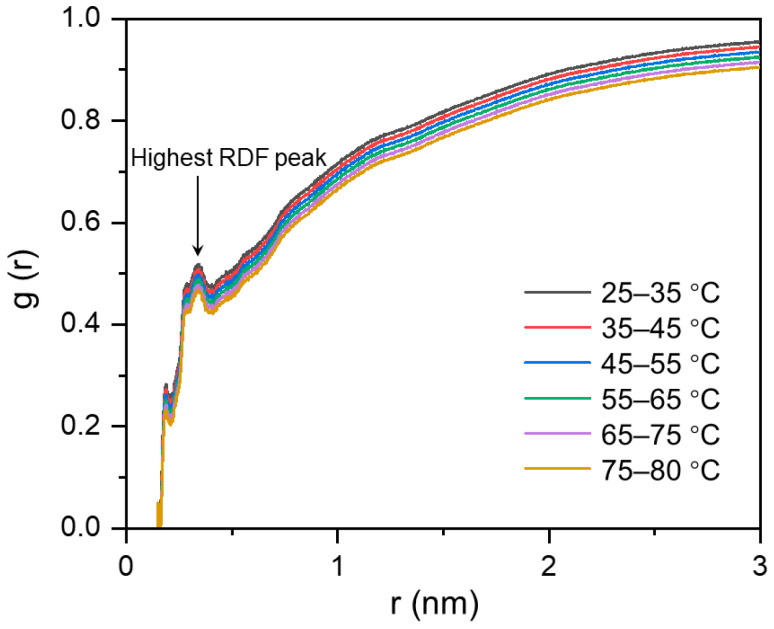
RDFs of the oxygen atoms of H_2_O molecules around the -OH groups of MPC-g-MC chains during the heating simulation process.

The radial distribution function (RDFs) of oxygen atoms on H_2_O molecules around the -OH groups on MPC-g-MC chains was also analyzed (Figure 4). A decrease in the highest RDF peak with rising temperature suggested that the tightly bound H_2_O molecules around the -OH groups on MPC-g-MC chains decreased. This result confirms the reduction of hydration shells and the strengthening of hydrophobic interactions during gelation.

**Figure 5:**
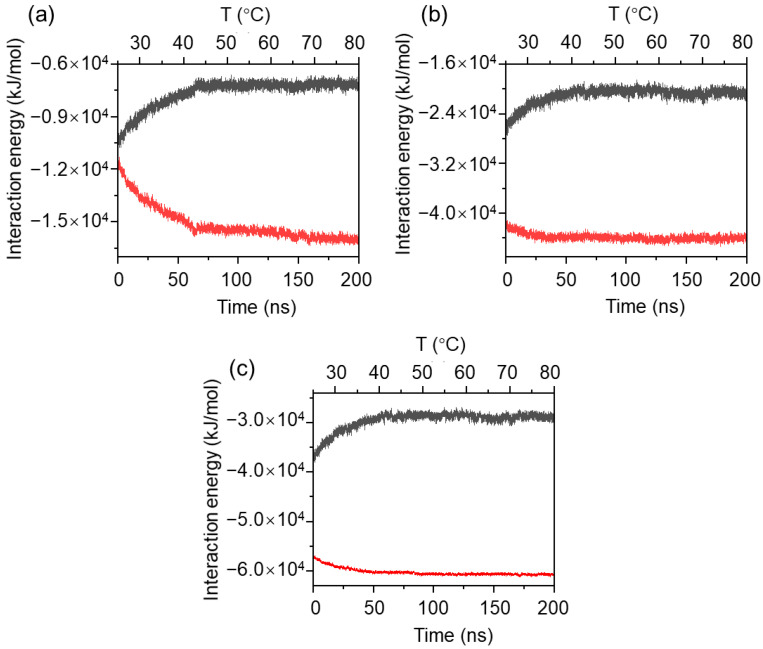
Interaction energies between MPC-g-MC chains and H_2_O molecules (black curve), and MPC-g-MC chains (red curve) during the heating simulation process: (**a**) Van der Waals energy, (**b**) Coulomb energy, (**c**) total interaction energy.

To investigate the effect of other non-bonded interactions on the thermosensitive gelation mechanism of the MPC-g-MC hydrogel, interaction energies, including Van der Waals and electrostatic energies, were analyzed as shown in Figure 5. All values of interaction energies were negative, implying that Van der Waals and electrostatic forces were attractive forces in the MPC-g-MC/H_2_O system. With increasing temperature, Van der Waals energy between MPC-g-MC chains enhanced (red curve in Figure 5a), while that between MPC-g-MC chains and H_2_O molecules weakened (black curve in Figure 5a). Similar trends appeared in the cases of Coulomb and total interaction energies (Figure 5b,c). These results further supported the temperature-driven aggregation mechanism.

In summary, the thermosensitive gelation mechanism of the MPC-g-MC hydrogel is related to changes in intermolecular interactions in the MPC-g-MC/H_2_O system. That is, the system’s energetic state and the balance of intermolecular interactions are altered as the temperature increases. During the heating process, the enhanced attractive interactions between MPC-g-MC chains, particularly hydrophobic interactions, and the weakened interactions between MPC-g-MC chains and H_2_O molecules are the fundamental causes of chain aggregation and subsequent gelation. Hydrophobic interaction is the primary driving force behind the aggregation of MPC-g-MC chains, while hydrogen bonding and Van der Waals forces synergistically promote the formation of a gel network.

The above simulation-based findings of the MPC-g-MC hydrogel are consistent with previously reported experimental studies on MPC-grafted hydroxypropyl cellulose (MPC-g-HPC) thermosensitive hydrogels, which employed techniques such as ^1^H NMR, FTIR, and differential scanning calorimetry (DSC) [40]. Those studies similarly indicated that a rise in temperature could enhance hydrophobic interactions among the MPC-g-HPC chains while destroying hydrogen bonding between MPC-g-HPC chains and H_2_O molecules, thereby inducing aggregation and gelation [40].

### 2.2. Aggregation Behavior and Intermolecular Interactions of MPC-g-MC Chains at Constant Temperatures

To further elucidate the thermosensitive gelation mechanism of MPC-g-MC hydrogels, MD simulations were performed under three constant temperature conditions: below, near, and above the gelation point, specifically at 25, 33 and 64 °C, respectively.

**Figure 6:**
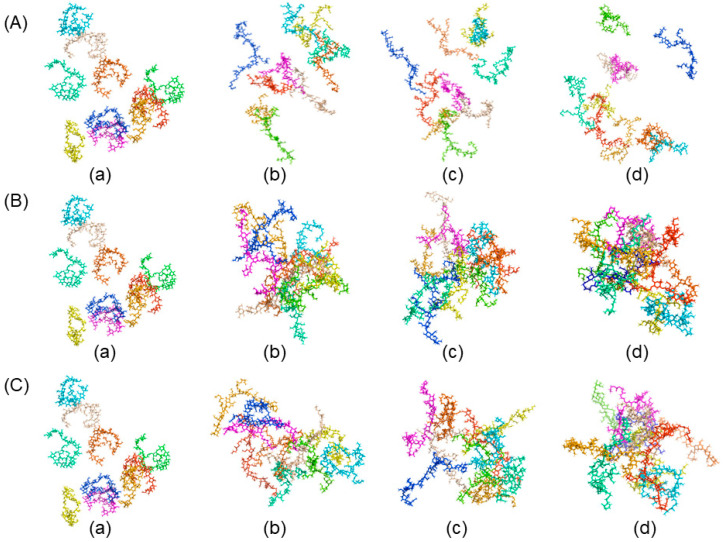
Conformations and aggregation behaviors of MPC-g-MC chains at different heating simulation temperatures and times (each MPC-g-MC chain was distinguished using different colors): (**A**) 25 °C, (**B**) 33 °C, (**C**) 64 °C; (**a**) 0 ns, (**b**) 50 ns, (**c**) 100 ns, (**d**) 200 ns.

Figure 6 shows the conformation and aggregation behaviors of MPC-g-MC chains at the above mentioned temperatures. The MPC-g-MC chains remained in a relatively dispersed and unentangled state throughout the simulation at 25 °C (Figure 6A). The MPC-g-MC chains entanglement and aggregation began to emerge within the first 50 ns at 33 °C, and this aggregated structure remained stable throughout the remaining simulation time (Figure 6B). A similar aggregation behavior was observed at 64 °C (Figure 6C). These results confirm that temperature has a significant impact on the conformation and aggregation behaviors of MPC-g-MC chains, with 33 °C identified as a critical threshold temperature, which is consistent with the experimentally measured gelation temperature (~33 °C) obtained from rheological testing [38].

**Figure 7:**
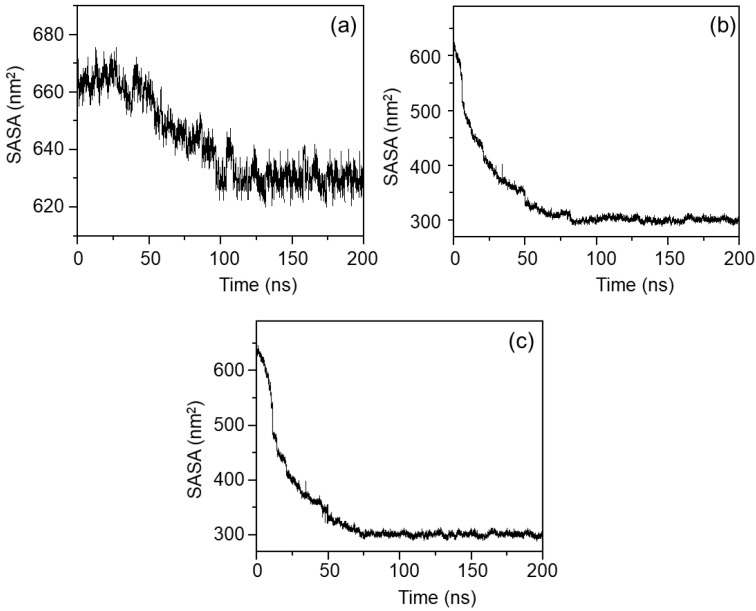
The relationship between the total SASA value of MPC-g-MC chains and simulation time at different temperatures: (**a**) 25 °C, (**b**) 33 °C, (**c**) 64 °C.

The total SASA value of MPC-g-MC chains at the three temperatures is presented in Figure 7. The overall SASA reductions (the differences of SASA values between 0–200 nm) were approximately 40, 300 and 315 nm^2^ at 25, 33 and 64 °C, respectively. These results suggested a minimal conformational change and the absence of aggregation of MPC-g-MC chains at 25 °C, while the MPC-g-MC chains progressively aggregated at 33 and 64 °C, which agrees with conformations and aggregation behaviors as shown in Figure 6. Additionally, equilibrium SASA values at the three temperatures were approximately 625 nm^2^ (25 °C), 312 nm^2^ (33 °C), and 310 nm^2^ (64 °C), indicating that dispersed MPC-g-MC chains expose more surface area to the H_2_O solvent at 25 °C.

**Figure 8:**
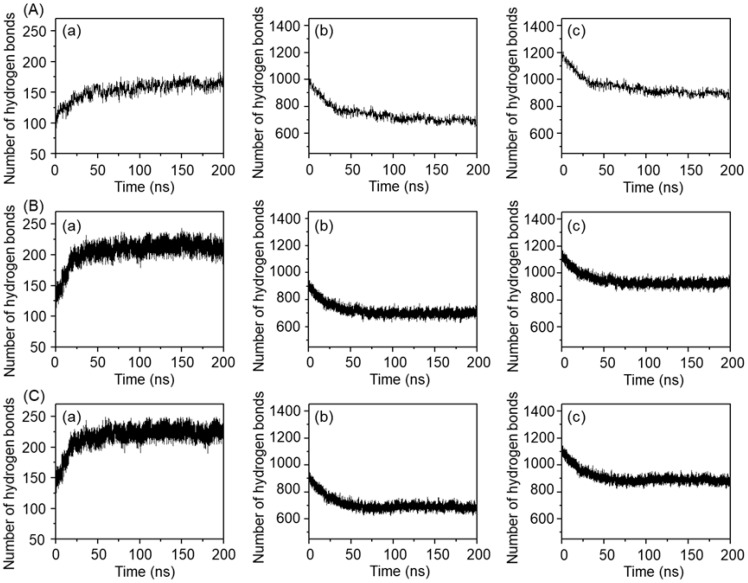
The relationship between the number of hydrogen bonds and simulation time in the MPC-g-MC/H_2_O system at (**A**) 25 °C, (**B**) 33 °C, (**C**) 64 °C; (**a**) hydrogen bonds between MPC-g-MC chains, (**b**) hydrogen bonds between MPC-g-MC chains and H_2_O molecules, (**c**) total hydrogen bonds in the MPC-g-MC/H_2_O system.

Hydrogen bonding behavior under the three temperature conditions is shown in Figure 8. During the simulation process, the number of hydrogen bonds between MPC-g-MC chains increased by ~60 at 25 °C (Figure 8(Aa)), while they increased by ~100 at 33 °C and 64 °C (Figure 8(Ba,Ca)). On the other hand, the number of hydrogen bonds between MPC-g-MC chains and H_2_O molecules decreased by ~200 at 25 °C (Figure 8(Ab)), while they decreased by ~270 at 33 °C and 64 °C (Figure 8(Bb,Cb)), resulting in an overall reduction in total hydrogen bonds (Figure 8(Ac,Bc,Cc)). These results indicate that a rise in temperature may promote hydrogen bond formation between MPC-g-MC chains while weakening hydration, thereby facilitating gelation.

**Figure 9:**
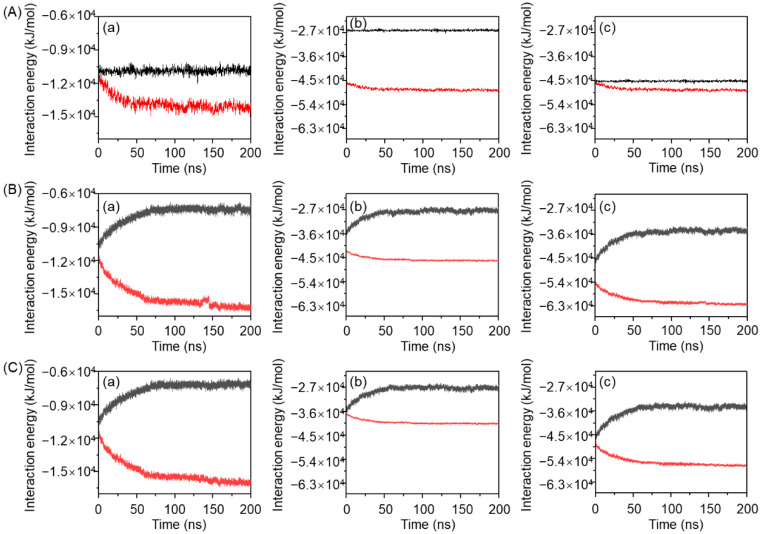
Interaction energies between MPC-g-MC chains and H_2_O molecules (black curve), and MPC-g-MC chains (red curve) during the simulation process: (**A**) 25 °C, (**B**) 33 °C, (**C**) 64 °C; (**a**) Van der Waals energy, (**b**) Coulomb energy, (**c**) total interaction energy.

Figure 9 illustrates that the interaction energies were negative at three temperatures in the MPC-g-MC/H_2_O system, further confirming that the Van der Waals and electrostatic forces were attractive forces. The relatively steady changes in the Van der Waals and Coulomb energies as the simulation time advanced resulted in the total interaction energy between MPC-g-MC chains being approximately equal to that between the MPC-g-MC chains and H_2_O molecules at 25 °C (Figure 9A). This result indicates a dynamic equilibrium between MPC-g-MC interchain attraction and MPC-g-MC/H_2_O solvent affinity. Such equilibrium could suppress MPC-g-MC chain aggregation and prevent gel network formation, thereby maintaining the system in a dynamically stable sol state.

In contrast, the interaction energies, especially Van der Waals energy, changed obviously with the simulation time at 33 °C and 64 °C (Figure 9(Ba,Bb,Ca,Cb)). As a result, the total interaction energies between MPC-g-MC chains increased (red curves in Figure 9(Bc,Cc)), while those between MPC-g-MC and H_2_O molecules decreased obviously (black curves in Figure 9(Bc,Cc)). These results imply that the aggregation of MPC-g-MC chains could happen at temperatures equal to or above the gelation temperature, while the binding affinity of the MPC-g-MC chains with H_2_O molecules decreases. Both of these mechanisms may facilitate the formation of the stable gel structure.

Analyses of the aggregation behavior and intermolecular interactions of MPC-g-MC chains at three specific temperatures further confirmed that temperature plays an important role in triggering the sol–gel transition of the MPC-g-MC hydrogel. At 33 °C (close to the gelation temperature of the MPC-g-MC hydrogel), the chain conformations of MPC-g-MC changed markedly, and the MPC-g-MC interchain interactions (especially hydrophobic forces) increased, while the interactions between MPC-g-MC chains and H_2_O molecules decreased, ultimately driving macroscopic gel formation [38].

### 2.3. Mechanism of Gelation Temperature Modulation by Phosphorylcholine Groups

**Figure 10:**
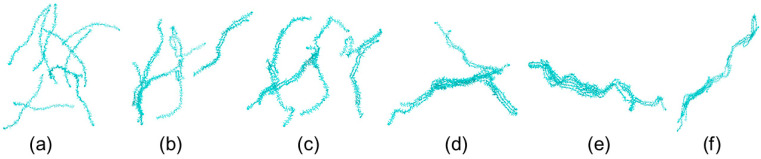
Conformations and aggregation behavior of MC chains during the heating simulation process: (**a**) 25 °C, (**b**) 30 °C, (**c**) 40 °C, (**d**) 55 °C, (**e**) 64 °C, (**f**) 80 °C.

To investigate the role of MPC groups in modulating the gelation temperature of the MPC-g-MC hydrogel, MD simulations were performed on the MC/H_2_O system. The conformation and aggregation behaviors of MC chains during the heating simulation process are shown in Figure 10. The results showed that MC chains remained dispersed at lower temperatures (Figure 10a–c). The MC chains began to aggregate at 55 °C (Figure 10d) and formed a stable aggregation at 64 °C (Figure 10e). The gelation temperature was thus determined to be in the range of 55–64 °C, which is consistent with experimental gelation temperatures reported in the literature (~60 °C) [41].

Such aggregation behavior and intermolecular interactions of MC chains were further investigated by using MD simulations at three specific temperatures (Figure S2), in which 25, 64 and 80 °C represented temperatures below, near, and above, respectively, the gelation temperature of the MC hydrogel. The MC chains remained in a dispersed and unentangled state at 25 °C during the simulation process (Figure S2A). The entangled and aggregated MC chains appeared at 64 °C when the simulation time was longer than 50 ns, and this aggregated structure remained stable throughout the remaining simulation time (Figure S2B). A similar aggregation behavior of MC chains was observed at 80 °C (Figure S2C). These results proved that the conformation and aggregation behaviors of MC chains are strongly dependent on temperature, showing a thermosensitive gelation characteristic.

The gelation mechanism of the MC hydrogel was investigated using MD simulations (Supplementary Materials). The results indicated that the gelation of MC is driven by changes in inter-molecular interactions with increasing temperature, similar to the case of the MPC-g-MC/H_2_O system. The rise in temperatures leads to stronger MC interchain interactions and weaker interactions between MC chains and H_2_O molecules. Among these interactions, enhanced hydrophobic interactions were identified as the primary driving force for MC chain aggregation and gelation (Figures S3 and S4), while increased MC interchain hydrogen bonding and synergistic Van der Waals and electrostatic interactions further promoted the sol–gel transition (Figures S5 and S6).

The simulation-derived gelation temperature of MPC-g-MC (~33 °C) was significantly lower than that of MC (55–64 °C). This observation agrees with previous experimental findings for MPC-g-HPC systems studied via ^1^H NMR, FTIR, and DSC, where MPC modification was found to reduce the gelation temperature by destroying the hydrogen bonding between MPC-g-HPC chains and H_2_O molecules [40].

**Figure 11:**
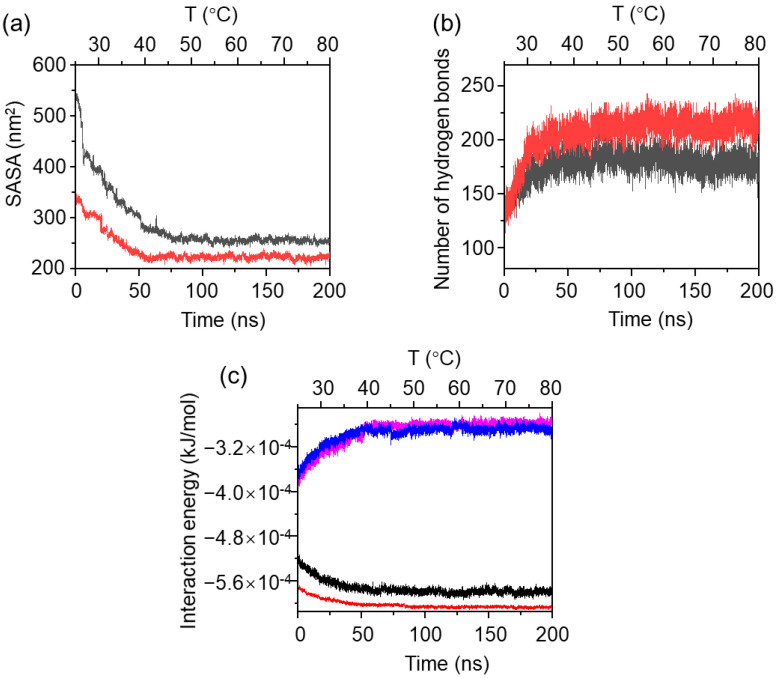
Comparative analyses of simulative interaction parameters of the MPC-g-MC/H_2_O and MC/H_2_O systems during the heating process: (**a**) hydrophobic SASA of MPC-g-MC chains (red curve) and MC chains (black curve), (**b**) number of hydrogen bonds between MPC-g-MC chains (red curve) and MC chains (black curve), (**c**) total interaction energies in two systems (pink curve: MPC-g-MC chains and H_2_O molecules; blue curve: MC chains and H_2_O molecules; black curve: MC chains; red curve: MPC-g-MC chains).

Figure 11 illustrates comparative analyses of simulative interaction parameters of the MPC-g-MC/H_2_O and MC/H_2_O systems. The reduction in hydrophobic SASA of MPC-g-MC chains was more pronounced than that of MC chains (Figure 11a), suggesting that the side MPC groups may enhance the hydrophobic interactions of the MPC-g-MC chains, thereby promoting their aggregations. The number of hydrogen bonds between MPC-g-MC chains was higher than that between MC chains (Figure 11b), implying that the side MPC groups could also promote aggregations of MPC-g-MC chains by increasing their interchain hydrogen bonding. Analysis of the total interaction energy indicated that the total attractive interactions (including Van der Waals and Coulomb forces) between MPC-g-MC chains and H_2_O were similar to those between MC chains and H_2_O (Figure 11c, pink and blue curves). However, it should be noted that the total attractive interactions between MPC-g-MC chains are stronger than those between MC chains (Figure 11c, red and black curves), further providing evidence that the side MPC groups enhance intermolecular forces, which support the formation of a stable MPC-g-MC gel network.

In summary, the MD simulation results demonstrated that the side MPC groups could enhance interactions between MPC-g-MC chains in the MPC-g-MC/H_2_O system, when compared with the MC/H_2_O system. As a result, the MPC-g-MC hydrogel exhibits a lower gelation temperature than that of the MC hydrogel.

## 3. Conclusions

Our works revealed the thermosensitive gelation mechanism of MPC-g-MC hydrogel and explained why its gelation temperature was lower than that of MC hydrogel at the molecular level using MD simulations. The MPC-g-MC/H_2_O system underwent a sol–gel transition at approximately 33 °C. The rise in temperature enhanced the hydrophobic interactions between MPC-g-MC chains and reduced interactions between MPC-g-MC chains and H_2_O molecules, accompanied by the reorganization of the hydrogen bonding network, bringing about aggregations of MPC-g-MC chains and the formation of the MPC-g-MC hydrogel. In comparison, MC exhibited gelation at a higher temperature (55–64 °C). The presence of the side MPC groups enhanced interactions between the MPC-g-MC chains, thus effectively lowering the gelation temperature of MPC-g-MC. These results provide a valuable theoretical insight for the investigation of novel thermosensitive MC-based hydrogels with gelation behavior near body temperature in the future.

## 4. Materials and Methods

### 4.1. Construction of the Model Molecule of MPC-g-MC

**Figure 12:**
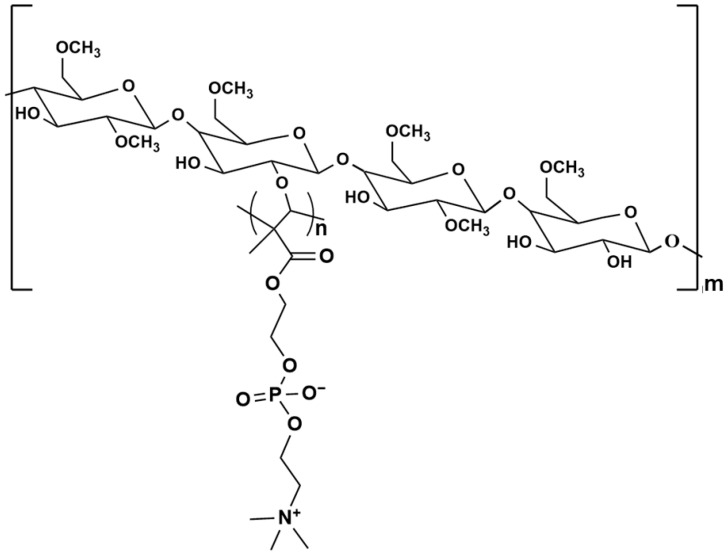
Chemical structure of MPC-g-MC model molecule (n = 6, m = 8).

The model molecule of MPC-g-MC was constructed as shown in Figure 12, according to its chemical structure [38]. The degrees of substitution of -CH_3_ and MPC groups were set at 1.5 and 0.25 in each repeating unit, respectively. Additionally, the number of repeating units of the backbone (m) was set to 8, and the number (n) in the MPC side chains was set to 6 (Supplementary Materials).

### 4.2. System Setup of MPC-g-MC/H_2_O Simulation

The mass concentration of MPC-g-MC in water was set at 10% based on our previous work [38]. A total of ten MPC-g-MC chains were inserted into the simulation box to construct the MPC-g-MC/H_2_O simulation system. The molecular structure and topological parameters of the system were constructed using CHARMM-GUI (https://charmm-gui.org/), accessed on 10 December 2024. [42]. The force field parameters for the MPC side chains were generated using the CGenFF online tool (https://cgenff.com/), accessed on 10 December 2024 [43]. Periodic boundary conditions (PBCs) were consistently applied throughout all simulations to reduce edge effects, maintain physical realism, and avoid artifacts arising from finite system size or self-interactions.

### 4.3. MD Simulation

All MD simulations were performed using the GROMACS 2022.3 software package [44]. GROMACS 2022.3 was used under the GNU General Public License (GPL), which ensures it is fully open-source and freely accessible to the research community. A citation to the software and a link to its source code (https://www.gromacs.org), accessed on 13 July 2024, have also been included. The CHARMM36 force field [45] combined with CGenFF [46] was employed to describe the interactions of MPC-g-MC molecules, while the TIP3P [47] water model was used for explicit solvation. The MPC-g-MC/H_2_O system was gradually heated from 298 K to 353 K (25–80 °C) using a single-step annealing approach during the heating simulation process. A time step of 2 femtoseconds was used for numerical integration with the Verlet algorithm. All covalent bonds involving hydrogen atoms were constrained using the LINCS algorithm. Long-range electrostatic interactions were computed using the particle-mesh Ewald (PME) method with a cutoff of 1.2 nm, which was also applied to Van der Waals interactions. The neighbor list was updated every 10 steps during the simulations.

Each simulation followed a standardized protocol, starting with energy minimization using the steepest descent algorithm for 10,000 steps to eliminate structural strain. This was followed by equilibration under constant volume and temperature (NVT ensemble) using the V-rescale thermostat, and subsequently under constant pressure and temperature (NPT ensemble) using the Berendsen’s method at 1 bar to equilibrate the system density. After a one-nanosecond equilibration phase, a 200-nanosecond production run was conducted, during which atomic coordinates were saved every 10 picoseconds for trajectory analysis.

The isothermal simulations were performed at 298 K (25 °C), 306 K (33 °C), and 337 K (64 °C), representing temperatures below, near, and above the gelation temperature of the MPC-g-MC hydrogel, respectively. The MD simulations were similar to the those used in the heating simulation process as mentioned above, except for removing the annealing setting.

### 4.4. Data Analysis

All molecular dynamic parameters were analyzed using built-in tools from the GROMACS 2022.3 package, along with visualization software including VMD 1.93 and PyMOL Molecular Graphics System (Version 2.0, Schrödinger LLC, New York, NY, USA) [48,49].

## Data Availability

All data are included the article and Supplementary Materials.

## Supplementary Materials

The following supporting information can be downloaded at: https://www.mdpi.com/article/10.3390/gels11070521/s1. Structural parameters of the model molecule of MPC-g-MC [38,40]. S2. Molecular dynamics simulation of methylcellulose (MC) hydrogel [38,50]. S3 Thermosensitive gelation mechanism of MC hydrogels. Figure S1. Chemical structure of MC model molecule (n = 16). Figure S2. Conformations and aggregation behaviors of MC chains at different heating simulation temperatures and times: (**A**) 25 °C, (**B**) 64 °C, (**C**) 80 °C; (**a**) 0 ns, (**b**) 50 ns, (**c**) 100 ns, (**d**) 200 ns. Figure S3. SASA values of MC chains during the heating simulation process and at different temperatures: (**A**) 25–80 °C, (**B**) 25 °C, (**C**) 64 °C and (**D**) 80 °C; (**a**) total SASA, (**b**) hydrophobic SASA, (**c**) hydrophilic SASA. Figure S4. RDFs of the oxygen atoms of H_2_O molecules around the -OH groups of MC chains during the heating simulation process. Figure S5. Number of hydrogen bonds in the MC/H_2_O system during the heating simulation process and at different temperatures: (**A**) 25–80 °C, (**B**) 25 °C, (**C**) 64 °C, (**D**) 80 °C; (**a**) hydrogen bonds between MC chains, (**b**) hydrogen bonds between MC chains and H_2_O molecules, (**c**) total hydrogen bonds in the MC/H_2_O system. Figure S6. Interaction energies between MC chains and H_2_O molecules (black curve), and MC chains (red curve) during the simulation process and at different temperatures: (**A**) 25–80 °C, (**B**) 25 °C (**C**) 64 °C, (**D**) 80 °C; (**a**) Van der Waals force, (**b**) Coulomb force, (**c**) total force.

## Abbreviations

The following abbreviations are used in this manuscript:

MPC	2-Methacryloyloxyethyl Phosphorylcholine
MC	Methylcellulose
MPC-g-MC	MPC-Grafted Methylcellulose
AGU	Anhydroglucose Unit
LCST	Lower Critical Solution Temperature
MD	Molecular Dynamics
SASA	Solvent Accessible Surface Area
RDF	Radial Distribution Function
VMD	Visual Molecular Dynamics
PME	Particle Mesh Ewald
LINCS	Linear Constraint Solver
NVT	Constant Number of particles, Volume, and Temperature
NPT	Constant Number of particles, Pressure, and Temperature
CHARMM	Chemistry at HARvard Macromolecular Mechanics
CGenFF	CHARMM General Force Field
TIP3P	Transferable Intermolecular Potential with 3 Points
DSC	Differential Scanning Calorimetry
FTIR	Fourier Transform Infrared Spectroscopy
^1^H NMR	Proton Nuclear Magnetic Resonance
SD Rat	Sprague–Dawley Rat
HPC	Hydroxypropyl Cellulose
PBC	Periodic Boundary Condition
