# Supplementary material for: Molecular Dynamics Simulation of the Thermosensitive Gelation Mechanism of Phosphorylcholine Groups-Conjugated Methylcellulose Hydrogel
Source: Gels. 2025 Jul 4;11(7):521. doi: 10.3390/gels11070521 (PMC12294316; doi:10.3390/gels11070521)
Supplement: Supplementary file 1 [file gels-11-00521-s001.zip › gels-3696029-supplementary.pdf]

Supplementary Materials

Molecular Dynamics Simulation of the Thermosensitive Gelation Mechanism of Phosphorylcholine Groups-conjugated Methylcellulose Hydrogel

Hongyu Mei, Yaqing Huang, Juzhen Yi, Wencheng Chen, Peng Guan, Shanyue Guan[2], Xiaohong Chen, Wei Li*, Liqun Yang*

S1. Structural Parameters of the Model Molecule of MPC-g-MC

In our previous work [1], the degree of substitution (DS) of MC groups of MC-g-MPC was 1.5, and that of MPC groups was 0.25. Consequently, the repetitive unit of the MC-g-MPC model compound was set to be composed of four AGU (anhydroglucose unit) residues (Figure 12), in which two AGU residues each carry two $–CH_3$ groups, and the other two AGU residues each carry one $–CH_3$ group. The MPC group is grafted onto one of the AGU residues that contains a single $–CH_3$ group. The number of repeating units (m) was set to 8.

Based on the literature [2], the number (n) in the MPC side chains of MPC-g-MC was calculated through Equation S1.

$$n = \frac{I_c/3}{I_{a+b}} \qquad \text{(S1)}$$

where I_c is the integral area of the trimethylammonium methyl protons of the MPC groups, and I_{a+b} is the integral area of the methoxy protons on the MC backbone in ^{1}H NMR spectrum of MPC-g-MC (i. e. MC-g-MPC-0.25 in our previous work) [1]. Accordingly, the n value was determined to be approximately 6.

S2. Molecular Dynamics Simulation of Methylcellulose (MC) Hydrogel

S2.1 Construction of the MC Model Compound

Based on our previous work [1], the structure of the MC model molecule is shown in Figure S1. The degree of $-CH_3$ substitution set at 1.5 in each repeating unit, and the number of repeating units (n) was set to 16.

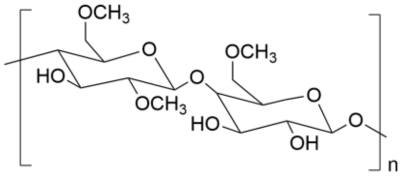

Figure S1. Chemical structure of MC model molecule (n=16).

S2.2 System Setup for MC Simulations

The concentration of MC in water was set at 10% (w/w) according to our previous work [1]. Ten MC chains were constructed in the MC/H_2O system. The model was generated using the GLYCAM modeling toolset (https://glycam.org) [3]. Periodic boundary conditions (PBCs) were consistently applied throughout all simulations to reduce edge effects, maintain physical realism, and avoid artifacts arising from finite system size or self-interactions.

S2.3 Molecular Dynamics Simulations

The MC/H_2O simulation system employed the GLYCAM06 force field parameters [3]. All other parameters were consistent with those used in the MPC-g-MC simulations. Isothermal simulations were conducted at 298 K (25 °C) (room temperature), 337 K (64 °C) (gelation temperature), and 383 K (64 °C) (high temperature), with the annealing step omitted. All remaining settings followed the protocols of the temperature-dependent simulations.

S2.4 Data Analysis

The analysis methods are the same as those for data analysis of the MPC-g-MC systems.

S3 Thermosensitive Gelation Mechanism of MC Hydrogel

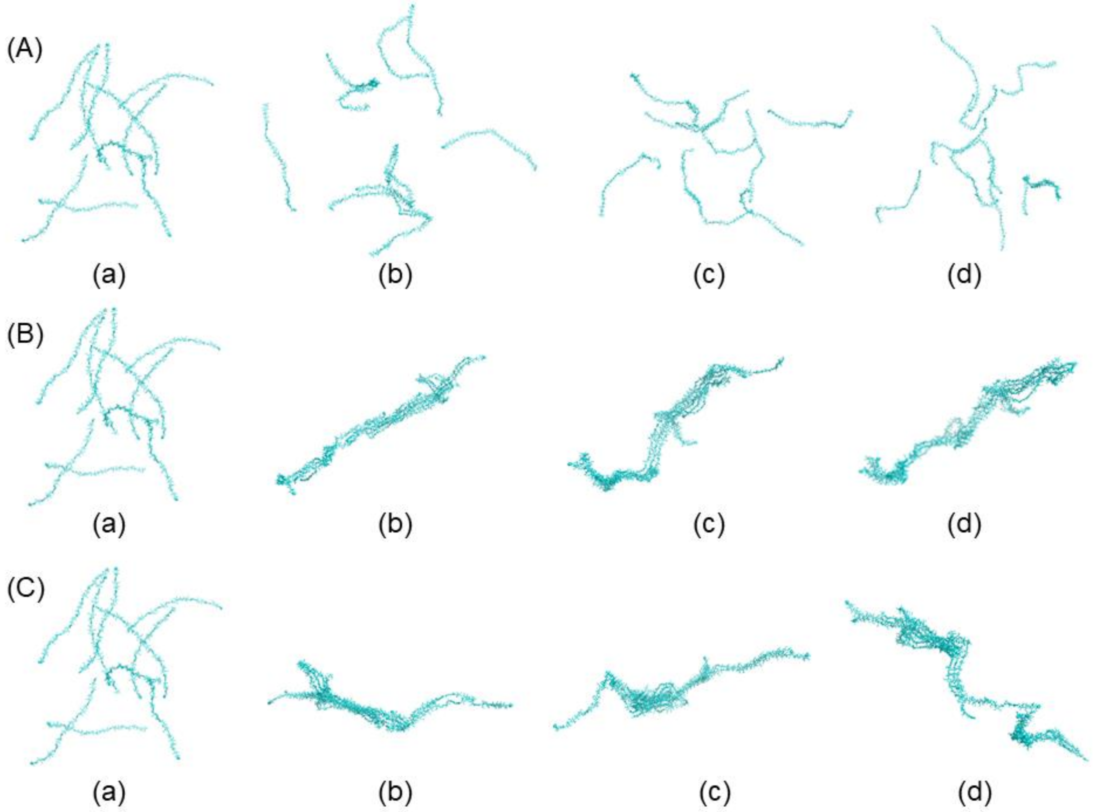

Figure S2. Conformations and aggregation behaviors of MC chains at different heating simulation temperatures and times: (A) 25 ºC, (B) 64 ºC, (C) 80 ºC; (a) 0 ns, (b) 50 ns, (c) 100 ns, (d) 200 ns.

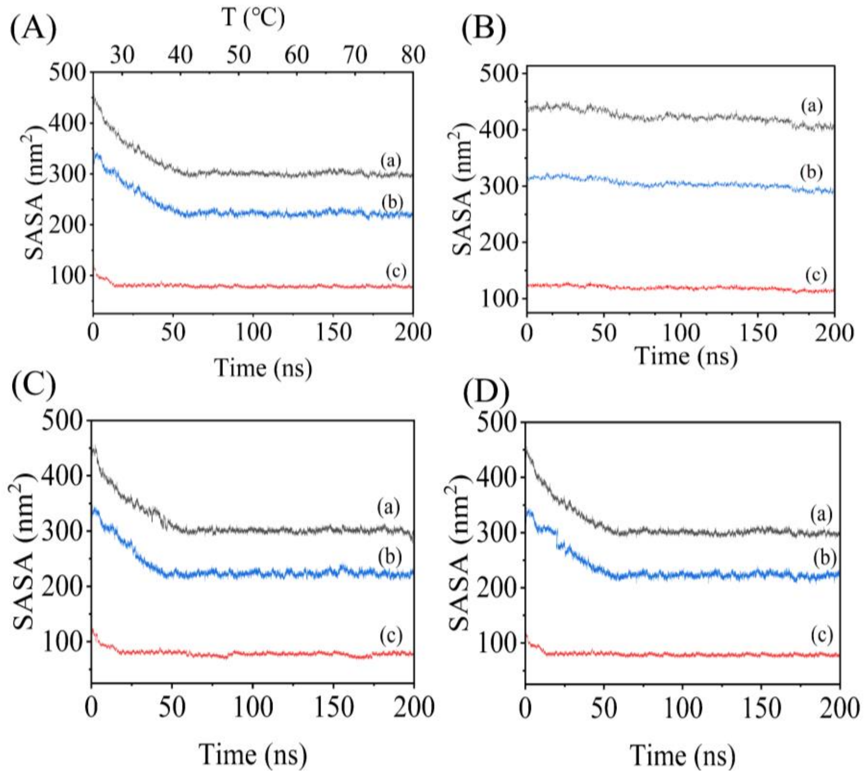

Figure S3. SASA values of MC chains during the heating simulation process and at different temperatures: (A) 25-80 °C, (B) 25 °C, (C) 64 °C and (D) 80°C; (a) total SASA (black curve), (b) hydrophobic SASA (blue curve), (c) hydrophilic SASA (red curve).

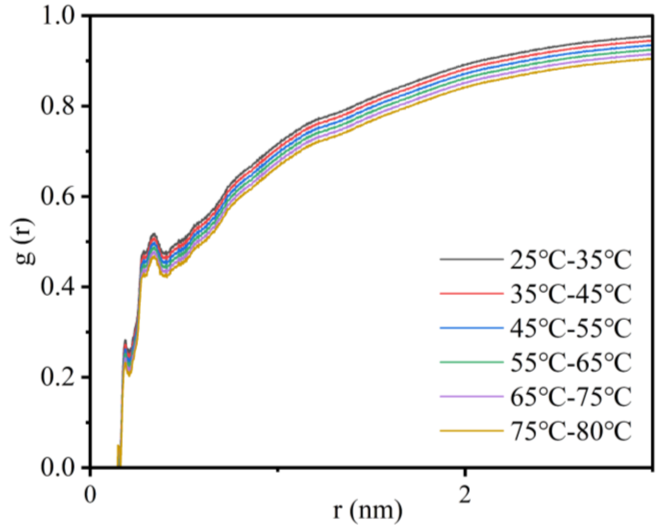

Figure S4. RDFs of the oxygen atoms of H_2O molecules around the -OH groups of MC chains during the heating simulation process.

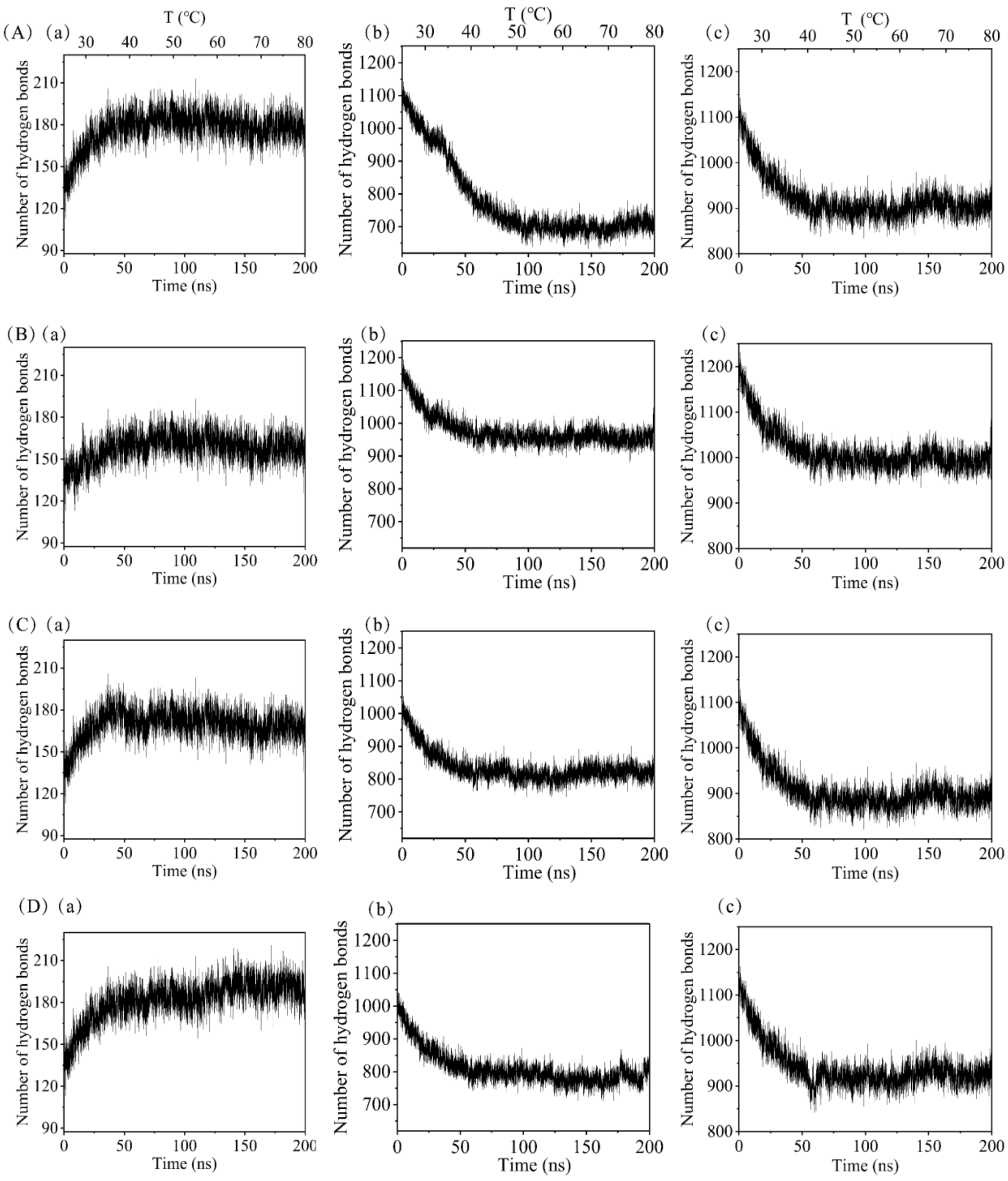

Figure S5. Number of hydrogen bonds in the MC/H_2O system during the heating simulation process and at different temperatures: (A) 25-80 ºC, (B) 25 ºC, (C) 64 ºC, (D) 80 ºC; (a) hydrogen bonds between MC chains, (b) hydrogen bonds between MC chains and H_2O molecules, (c) total hydrogen bonds in the MC/H_2O system.

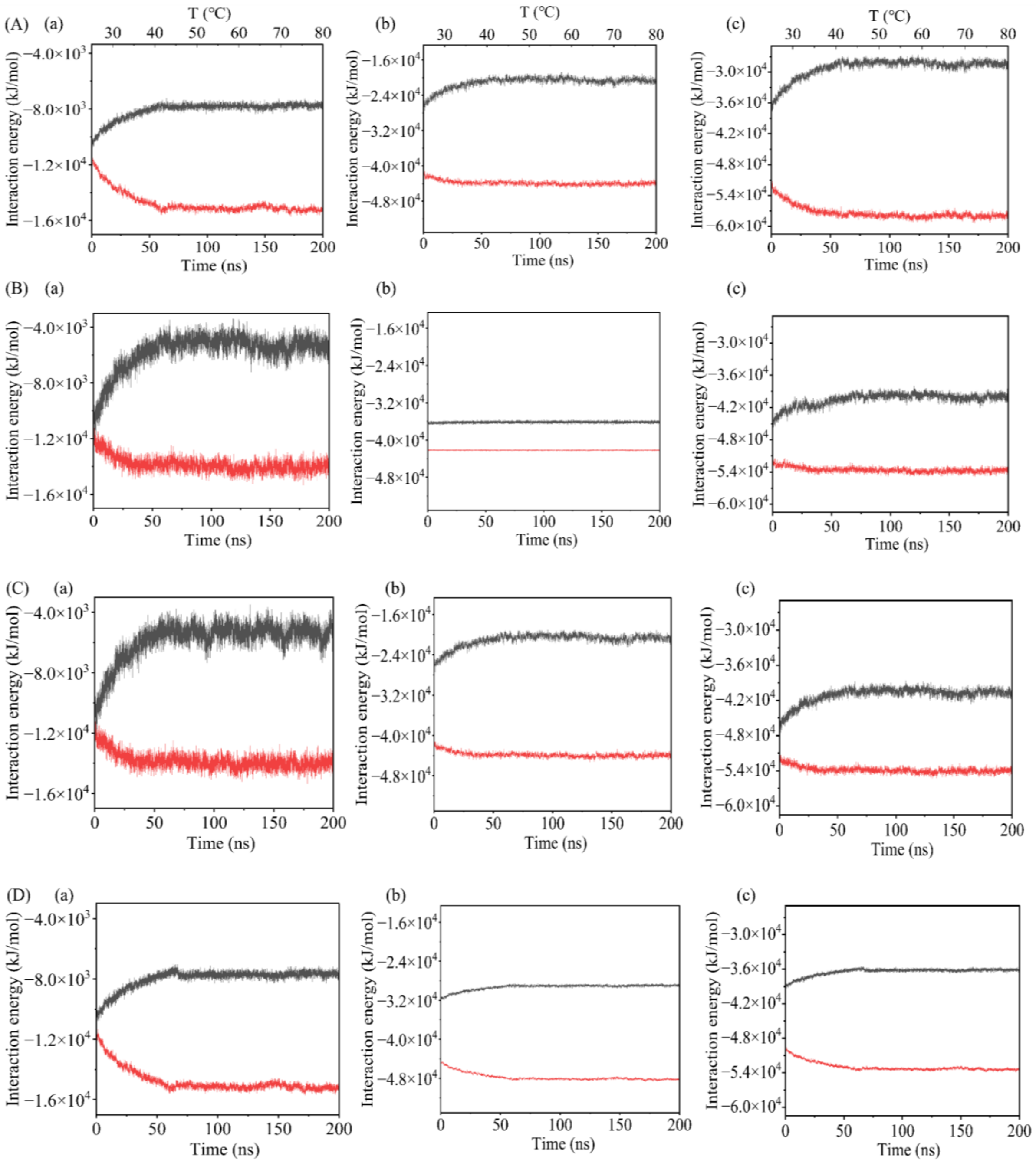

Figure S6. Interaction energies between MC chains and H_2O molecules (black curve), and MC chains (red curve) during the simulation process and at different temperatures: (A) 25-80 ºC, (B) 25 ºC (C) 64 ºC, (D) 80 ºC; (a) Van der Waals force, (b) Coulomb force, (c) total force.

References

[1] Huang, Y.; Guo, W.; Zhang, J.; Peng, X.; Li, G.; Zhang, L.-M.; Yang, L. Thermosensitive hydrogels based on methylcellulose derivatives for prevention of

postoperative adhesion. *Cellulose* **2020**, *27*, 1555-1571. doi:10.1007/s10570-019-02857-4.

[2] Ci, J.; Kang, H.; Liu, C.; He, A.; Liu, R. Thermal Sensitivity and Protein Anti-Adsorption of Hydroxypropyl Cellulose-g-Poly(2-(methacryloyloxy) Ethyl phosphorylcholine). *Carbohydrate Polymers* **2017**, *157*, 757-765, doi:https://doi.org/10.1016/j.carbpol.2016.10.051.

[3] Kirschner, K.N.; Yongye, A.B.; Tschampel, S.M.; González-Outeiriño, J.; Daniels, C.R.; Foley, B.L.; Woods, R.J. GLYCAM06: A generalizable biomolecular force field. Carbohydrates. *Journal of Computational Chemistry* **2008**, *29*, 622-655. doi: https://doi.org/10.1002/jcc.20820.
